# Accelerated free-breathing diffusion tensor MRI of the entire human heart using spatiotemporal registration and retrospective image selection

**DOI:** 10.1186/1532-429X-18-S1-O52

**Published:** 2016-01-27

**Authors:** Choukri Mekkaoui, Timothy G Reese, Marcel P Jackowski, Himanshu Bhat, David E Sosnovik

**Affiliations:** 1 Harvard Medical School - Massachussets General Hospital, Charlestown, MA USA; 2 University of São Paulo, São Paulo, Brazil; 3 Siemens, Boston, MA USA

## Background

Currently diffusion tensor MRI (DTI) of the heart can be performed *in vivo* either with a dual-gated STE [1] or gated motion-compensated PGSE [2]. Both are inefficient due to cardiac and respiratory motion leading to long scan times [3]. Simultaneous multi-slice (SMS) blipped-CAIPI [4] has emerged as a unique EPI technique to acquire multiple slices simultaneously. SMS has been used for cardiac DTI with breath-holding (BH) but with limited coverage [5]. Here, we present a clinically feasible whole-heart free-breathing (FB) accelerated DTI approach by combining SMS with *i)* sequential acquisition of all repetitions of each direction and *ii)* spatiotemporal registration (STR) followed by retrospective image selection, avoiding navigator echoes or controlled respiration. Our approach facilitates whole-heart coverage with scan time under 15 minutes, leading to reproducible DTI measurements, and enabling 3D tractography.

## Methods

DTI was performed in healthy volunteers (n = 7) on a clinical 3T scanner (Siemens Skyra), with an ECG-gated STE sequence. Acquisition parameters were: FOV = 360 × 200 mm^2^, resolution 2.5 × 2.5 mm^2^, thickness = 8 mm, in-plane GRAPPA rate 2, TE = 34 ms, b-values = 0 and 500s/mm^2^, 10 diffusion-encoding directions, 8 averages, and 12 contiguous short-axis slices. Rate 2 SMS excitation was followed by a blipped-CAIPI readout. A sequential acquisition of diffusion-encoding directions evenly distributes the rejections across all directions ensuring that we can select enough samples of each direction. STR was applied to reduce the misregistration resulting from respiratory motion [6]. Following STR, we utilize a novel entropy-based retrospective image selection method to reject corrupted images and maximize SNR. Mean diffusivity (MD), fractional anisotropy (FA) and helix angle (HA) values were compared between BH and FB.

## Results

**Figure 1:**
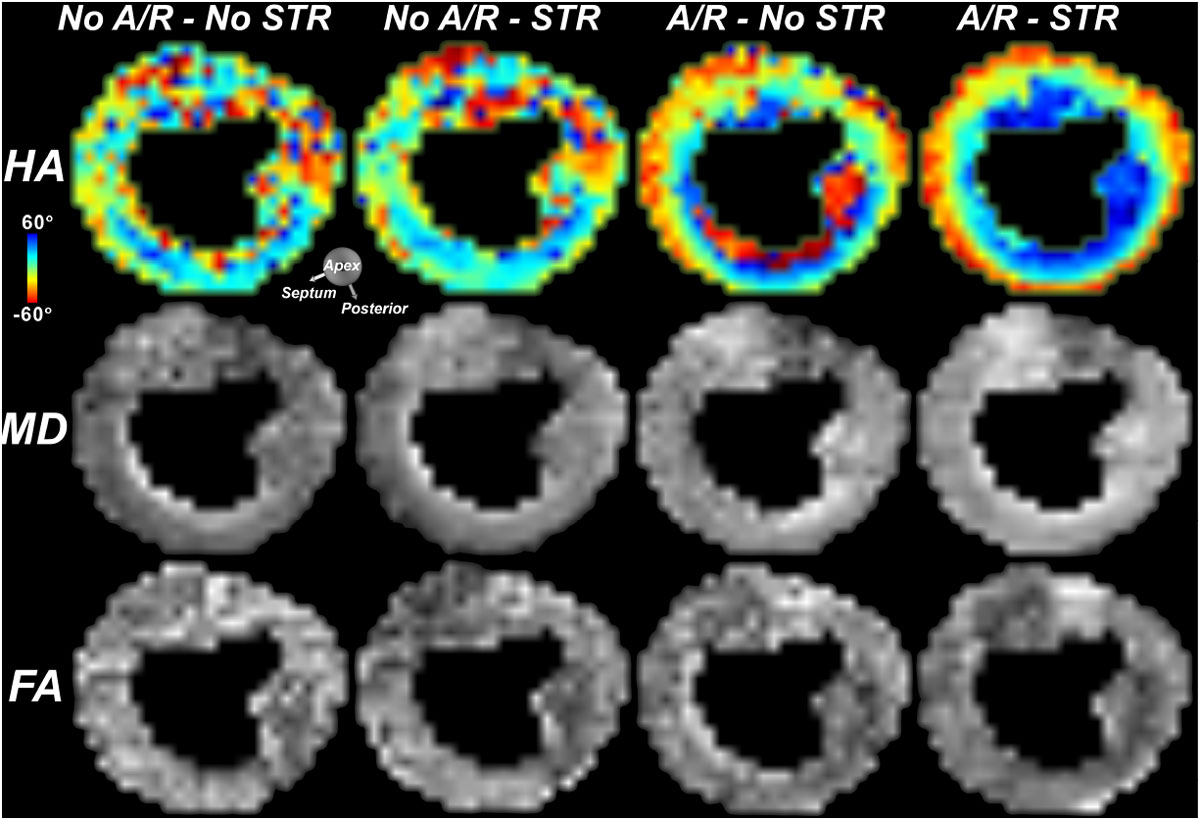
**Impact of spatiotemporal registration (STR) and retrospective image acceptance/rejection (A/R) on free-breathing DTI of the heart**. In the absence of both STR and A/R (first column) the HA, MD and FA maps are extremely noisy. The use of STR without A/R (second column) has little impact. A/R alone (third column) improves image quality substantially, but the maps remain noisy due to misregistration. In contrast, the use of STR followed by A/R (final column) produces high quality maps.

**Figure 2:**
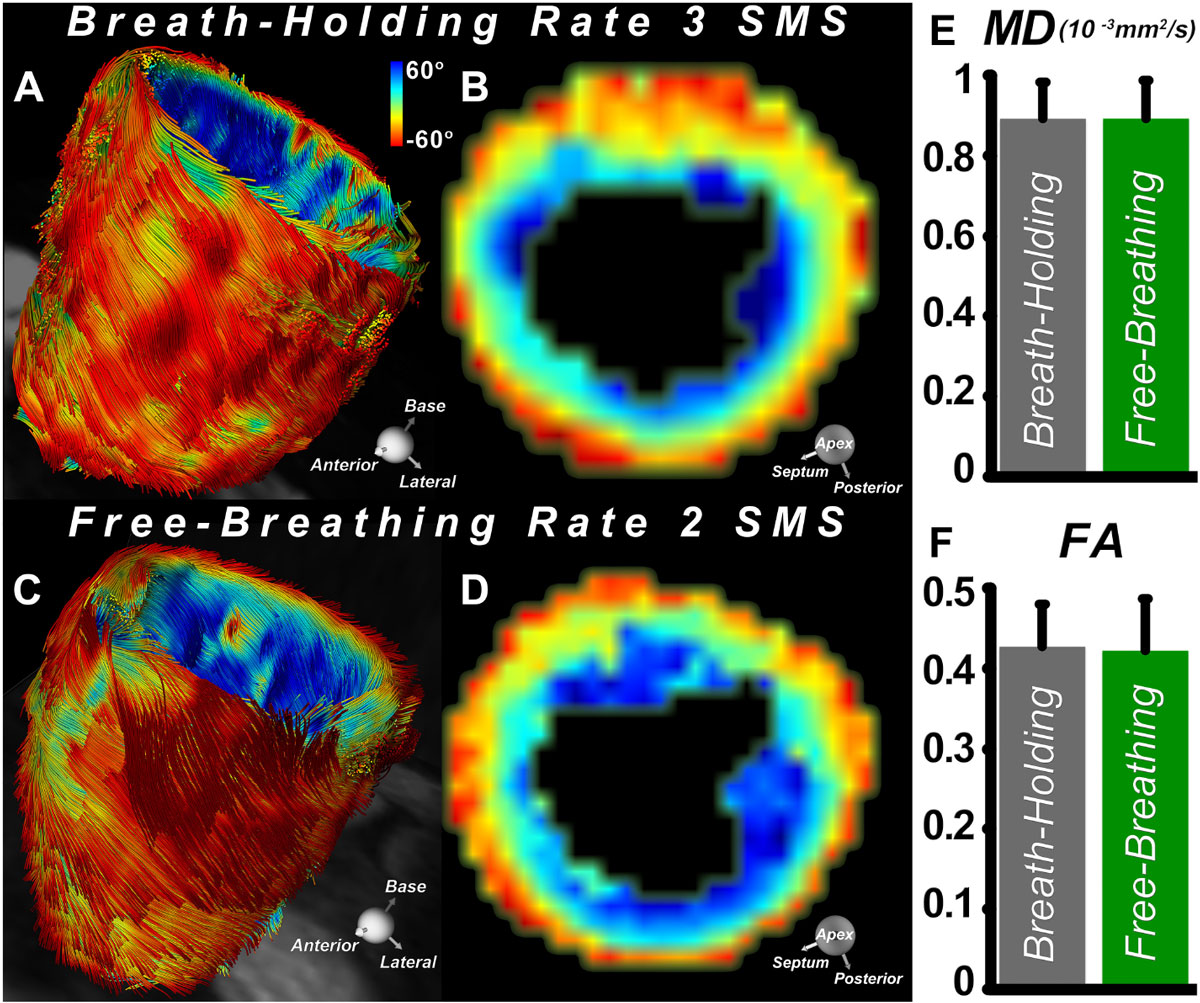
**Comparison of breath-hold (A, B) and free-breathing (C, D) DTI of the heart**. Tractography of the entire LV could be accurately performed using breath hold DTI with rate 3 SMS (**A**), and free-breathing DTI with rate 2 SMS (**C**). The quality of HA maps derived from breath-hold (**B**) and free-breathing (**D**) DTI datasets compared extremely well. (**E**, **F**) No significant differences were seen in HA maps or MD and FA indices between the breath-hold and free-breathing approaches.

Accelerated FB DTI using SMS was performed in all 7 volunteers with the proposed sequential gradient acquisition order. Figure 1 demonstrates how the addition of STR and retrospective image selection improves quality of HA, MD, and FA. Tractography of the entire LV was performed using both BH rate 3 SMS and FB rate 2 SMS. No significant differences in HA maps or MD and FA were seen between the FB and BH (Figure 2). The entire LV was imaged during FB under 15 minutes.

## Conclusions

We introduce a clinically feasible whole-heart accelerated FB DTI with scan time under 15 minutes. The results compare favorably with those previously acquired and validated using BH. Future improvements could include increased SMS acceleration resulting from advanced purpose-built RF coils. The integration of DTI into clinical protocols may enable the reliable characterization of myocardial structure in patients with cardiac diseases.
